# Regulation of Hippo-YAP signaling by insulin-like growth factor-1 receptor in the tumorigenesis of diffuse large B-cell lymphoma

**DOI:** 10.1186/s13045-020-00906-1

**Published:** 2020-06-16

**Authors:** Xiangxiang Zhou, Na Chen, Hongzhi Xu, Xiaoming Zhou, Jianhong Wang, Xiaosheng Fang, Ya Zhang, Ying Li, Juan Yang, Xin Wang

**Affiliations:** 1 Department of Hematology, Shandong Provincial Hospital Affiliated to Shandong University, No.324, Jingwu Road, Jinan, 250021 Shandong China; 2 Department of Science and Education, Shandong Provincial Hospital Affiliated to Shandong University, Jinan, 250021 Shandong China; 3 School of Medicine, Shandong University, Jinan, 250012 Shandong China; 4 Shandong Provincial Engineering Research Center of Lymphoma, Jinan, 250021 Shandong China; 5 Key Laboratory for Kidney Regeneration of Shandong Province, Jinan, 250021 Shandong China

**Keywords:** DLBCL, Hippo-YAP, IGF-1R, Verteporfin, CRISPR/Cas9

## Abstract

**Background:**

Hippo-Yes-associated protein (YAP) signaling is a key regulator of organ size and tumorigenesis, yet the underlying molecular mechanism is still poorly understood. At present, the significance of the Hippo-YAP pathway in diffuse large B-cell lymphoma (DLBCL) is ill-defined.

**Methods:**

The expression of YAP in DLBCL was determined in public database and clinical specimens. The effects of YAP knockdown, CRISPR/Cas9-mediated YAP deletion, and YAP inhibitor treatment on cell proliferation and the cell cycle were evaluated both in vitro and in vivo. RNA sequencing was conducted to detect dysregulated RNAs in YAP-knockout DLBCL cells. The regulatory effects of insulin-like growth factor-1 receptor (IGF-1R) on Hippo-YAP signaling were explored by targeted inhibition and rescue experiments.

**Results:**

High expression of YAP was significantly correlated with disease progression and poor prognosis. Knockdown of YAP expression suppressed cell proliferation and induced cell cycle arrest in DLBCL cells. Verteporfin (VP), a benzoporphyrin derivative, exerted an anti-tumor effect by regulating the expression of YAP and the downstream target genes, CTGF and CYR61. In vitro and in vivo studies revealed that deletion of YAP expression with a CRISPR/Cas9 genome editing system significantly restrained tumor growth. Moreover, downregulation of IGF-1R expression led to a remarkable decrease in YAP expression. In contrast, exposure to IGF-1 promoted YAP expression and reversed the inhibition of YAP expression induced by IGF-1R inhibitors.

**Conclusions:**

Our study highlights the critical role of YAP in the pathogenesis of DLBCL and uncovers the regulatory effect of IGF-1R on Hippo-YAP signaling, suggesting a novel therapeutic strategy for DLBCL.

## Introduction

Diffuse large B-cell lymphoma (DLBCL) is the most common subtype of lymphoma, accounting for 25~35% of non-Hodgkin lymphoma (NHL) cases [1]. With the advent of immunochemotherapy based on rituximab, remarkable progress has been made in the treatment of DLBCL. However, 30~40% of patients still present relapsed/refractory disease with poor response rates for salvage therapy [2]. DLBCL is characterized by high heterogeneity in gene expression profiles and the clinical course [3]. Aberrations in gene expression result in altered activation of signaling pathways and variations in therapeutic responses [4, 5]. It remains largely unknown whether and how these genetic and signaling alterations contribute to lymphomagenesis, and further investigations on novel therapeutic targets are still warranted.

Hippo-Yes-associated protein (YAP) signaling was originally discovered in *Drosophila*. It is an evolutionarily conserved growth-control pathway that plays fundamental roles in governing organ size and cell proliferation [6]. The transcriptional coactivator YAP is the major downstream effector of the core kinase cascade in this signaling pathway [7]. The Hippo kinase cascade in vertebrates is composed of MST1/2, LATS1/2, and their adaptor proteins [8]. Activation of MST1/2 leads to phosphorylation of the growth-promoting transcriptional coactivator YAP, facilitates YAP degradation in the cytoplasm, and further inhibits the interaction of YAP with TEAD, resulting in the activation of downstream targets (CTGF and CYR61) [9]. Given the Hippo-YAP signaling does not have a unique extracellular “Hippo-specific” ligand or a specific membrane receptor, its activation mainly depends on crosstalk mechanisms [10].

In recent years, the Hippo-YAP signaling pathway has emerged as a crucial player in the development of human malignancies [11–13]. As a key effector of this signaling pathway, YAP functions as a transcriptional coactivator in modulating cell growth, cell apoptosis, and drug resistance in several human malignancies [13, 14]. Recent studies have suggested that the function of YAP in cancer cells is cell-type and/or cellular-context dependent. YAP interacts with extracellular signaling to induce the development and progression of cervical cancer [15, 16]. Moreover, YAP was also reported to act as a tumor suppressor in certain conditions. It was demonstrated that YAP could enhance p73-dependent and ABL1-induced cell apoptosis during the DNA-damage process [17, 18]. Hence, a better understanding of Hippo-YAP signaling will facilitate the prevention and treatment of cancer. At present, the biological function and underlying mechanism of Hippo-YAP signaling in DLBCL are still undefined.

Herein, we described for the first time the expression pattern and prognostic significance of YAP in DLBCL. Furthermore, abrogation of YAP expression either by shRNA treatment or CRISPR/Cas9-mediated knockout attenuated the tumorigenic characteristics of DLBCL cells, causing cell proliferation inhibition and cell cycle arrest. Inhibition of insulin-like growth factor-1 receptor (IGF-1R) in DLBCL cells led to decreased YAP expression and subsequently restrained the activation of YAP downstream targets. Overall, this study highlights that targeting Hippo-YAP via IGF-1R may provide a novel therapeutic strategy for DLBCL treatment.

## Methods

### Patients and clinical samples

Lymph node samples from 60 de novo DLBCL patients and 30 reactive lymphoid hyperplasia patients were collected. Histological diagnoses were established according to the World Health Organization (WHO) classification [19]. Normal peripheral blood mononuclear cells (PBMCs) from healthy volunteers were isolated by the Ficoll-Hypaque density gradient centrifugation method (TBD Science, Tianjin, China). Normal B cells were purified from freshly isolated PBMCs using CD19+ magnetic microbeads kit (Miltenyi Biotec, Bergisch Gladbach, Germany) as previously reported [20, 21]. This study was approved by the Medical Ethical Committee of Shandong Provincial Hospital Affiliated to Shandong University. All samples were obtained with informed consent in accordance with the Declaration of Helsinki.

### Cell lines and reagents

Human DLBCL cell lines (LY1, LY8, LY3, and Val) were routinely cultured in Iscove’s modified Dulbecco’s medium (IMDM) with 10% heat-inactivated fetal bovine serum (Gibco, MD, USA). The medium contained a 1% penicillin/streptomycin mixture and 2 mM glutamine. Verteporfin (VP; a YAP inhibitor, SML0534) was obtained from Sigma (MO, USA). Recombinant human IGF-1 was obtained from PeproTech (100-11, NJ, USA). Doxorubicin (S1208), AG1024 (an IGF-1R inhibitor, S1234), and picropodophyllin (PPP; an IGF-1R inhibitor, S7668) were purchased from Selleckchem (TX, USA).

### Lentivirus-mediated knockdown of YAP expression

The sequences for YAP and IGF-1R shRNAs were as follows: shYAP 1#, 5′-GCCACCAAGCTAGATAAAGAA-3′; shYAP 2#, 5′-CCCAGTTAAATGTTCACCAAT-3′. shIGF-1R 1#, 5′-GCCGAAGATTTCACAGTCAAA-3′; and shIGF-1R 2#, 5′-GCCTTTCACATTGTACCGCAT-3′. The control shRNA was synthesized with a scrambled sequence. The shRNAs were cloned into lentiviral vectors (GeneChem, Shanghai, China). Lentivirus infection was carried out according to the manufacturer’s instruction. Stably transfected cells were selected with puromycin (2.0 μg/ml). Seventy-two hours after transfection, the cells were collected and used for subsequent analysis.

### CRISPR/Cas9-mediated generation of YAP-knockout cells

YAP-knockout (YAP^−/−^) cells were generated using CRISPR/Cas9 genomic editing system. The production and packaging of lentiviral vectors for stably expressing Cas9-gRNA was accomplished by GeneChem. The gRNA target sites for YAP deletion were as follows: sgYAP#1, 5′-TGGGGGCTGTGACGTTCATC-3′; sgYAP#2, 5′-GAGCACTCTGACTGATTCTC-3′; and sgYAP#3, 5′-ACATCGATCAGACAACAACA-3′. Validation of YAP^−/−^ cells selected by puromycin (2.0 μg/ml) was conducted by PCR analysis of genomic DNA coupled with DNA sequencing. The primers to amplify sgYAP cut sites are listed in Table S1.

### Immunohistochemistry and hematoxylin-eosin staining

Immunohistochemical staining was performed as previously described [20]. The negative control was detected with the primary antibody replaced by PBS. Immunohistochemical staining was scored by two independent observers who were blinded to the patients’ clinical data. The scoring system for grading the level of YAP was reported previously [22]. The expression level was evaluated by immunohistochemistry (IHC) score calculated by multiplying a proportion score and intensity score. The proportion score reflected the fraction of positive-stained cells (0, none; 1, ≤ 10%; 2, 10–25%; 3, 25–50%; and 4, > 50%), and the intensity score revealed the staining intensity (0, no staining; 1, weak; 2, intermediate; and 3, strong). Finally, the total score was calculated. High and low protein expression levels were defined using the mean score of all samples as a cutoff point. With these criteria, tissue staining could be interpreted as “low” or “high.” The primary antibodies used were anti-YAP (Proteintech Group, 13584-1-AP, IL, USA), anti-Ki67 (Proteintech Group, 27309-1-AP), and anti-c-myc (Abcam, ab32072, Cambridge, UK). For hematoxylin-eosin (H&E) staining, fresh subcutaneous tumors isolated from mice were fixed in 4% paraformaldehyde (PFA) and embedded in paraffin for histological examinations. Sections with thickness of 4 μm were cut and stained with H&E.

### Nuclear and cytoplasmic fractionation

Nuclear and cytoplasmic proteins were isolated with NE-PER Nuclear and Cytoplasmic Extraction Reagents (Thermo Fisher Scientific, MA, USA) following the manufacturer’s instructions. The levels of GAPDH and Histone H3 were used as loading controls for the nuclear and cytoplasmic fractions, respectively

### Western blot analysis

Cell lysates were extracted with radioimmunoprecipitation assay buffer together with a 1× phosphatase inhibitor cocktail (PhosSTOP, Roche, Basel, Switzerland). Western blotting was performed as previously described [20, 23]. The primary antibodies used were anti-phospho-IGF-1R (Tyr1135/1136) (Cell Signaling Technology, 3024, MA, USA), anti-IGF-1R (Cell Signaling Technology, 9750), anti-Mcl-1 (Cell Signaling Technology, 5453), anti-Caspase-3 (Cell Signaling Technology, 14220), anti-cleaved Caspase-3 (Cell Signaling Technology, 9661), Caspase-8 (Cell Signaling Technology, 9746), anti-cleaved Caspase-8 (Cell Signaling Technology, 8592), anti-Histone H3 (Cell Signaling Technology, 4499), anti-YAP (Proteintech Group, 13584-1-A), anti-TEAD (Abcam, ab197589), anti-MST1 (Abcam, ab124787), anti-Bcl-XL (Abcam, ab32370), and anti-GAPDH (Zhongshan Goldenbridge, TA-09, Beijing, China).

### Cell proliferation assessment

Cell proliferation was measured with Cell Counting Kit-8 (CCK-8) method (Dojindo Laboratories, Kumamoto, Japan) as previously described [20]. DLBCL cells (1 × 10^4^ cells/100 μl/well) given the designated treatment were seeded in 96-well plates. Thereafter, the cells were incubated with 10 μl/well CCK-8 for 4 h according to the manufacturer’s protocol. Cell proliferation was detected by measuring light absorption at 450 nm with the SpectraMax M2 Microplate Reader (Molecular Devices, CA, USA).

### Analyses of cell apoptosis and the cell cycle

Both cell apoptosis assays and cell cycle assays were performed on Navios flow cytometer (Beckman Coulter, CA, USA). Cell apoptosis was detected by Annexin V-PE/7-AAD assay or Annexin V-FITC/ propidium iodide (PI) staining according to the manufacturer’s instructions (BD Biosciences, MA, USA). For cell cycle analysis, cells were washed with PBS, fixed with 70% ethanol overnight at 4 °C, and resuspended in PI/RNase staining solution (BD Biosciences). The percentage of cells in the indicated cell cycle phase was calculated with ModFit LT version 3.2 software.

### RNA-sequencing

RNA from YAP^−/−^ cells was prepared for RNA-sequencing (RNA-seq; three biological replicates for each group). RNA-seq experiments were performed by Novogene (Beijing, China). Briefly, total RNA was isolated with TRIzol reagent (Invitrogen, MA, USA). Sequencing libraries were generated following the manufacturer’s recommendations. After cluster generation, the library preparations were sequenced on an Illumina HiSeq platform. HTSeq v0.6.0 was applied to count the numbers of reads mapped to each gene, and the fragments per kilobase of transcript per million fragments mapped (FPKM) of each gene were calculated. Gene Ontology (GO) and Kyoto Encyclopedia of Genes and Genomes (KEGG) analyses were implemented using the cluster Profiler R package. A hierarchical clustering heat map was generated with the ggplot library.

### RNA isolation and quantitative real-time PCR

Total RNA was extracted with RNAiso Plus (TaKaRa, Dalian, China) and reverse transcribed into cDNA with PrimeScript RT reagent kit (TaKaRa). Amplification reactions were conducted with SYBR Green (TaKaRa) on LightCycler 480II real-time PCR system (Roche). The primers for quantitative real-time PCR (qRT-PCR) are listed in Table S2. Real-time PCR for each sample was performed in triplicate. Relative quantification was determined by means of the 2^−ΔΔ^^CT^ method with LightCycler 480 Gene Scanning version 1.5 software.

### Immunofluorescence assays

Cells given the designed treatment were seeded on glass slides in a liquid thin layer cell smear, fixed in 4% paraformaldehyde, permeabilized with 0.1% Triton X-100, and then blocked with 5% normal goat serum for 1 h at room temperature. The slides were incubated with primary antibodies at 4 °C overnight, followed by incubation for 1 h at room temperature with Dylight 488-conjugated goat anti-rat IgG antibody (Abbkine, Beijing, China). The slides were washed with PBS and mounted with DAPI. Images were acquired with Pannoramic DESK Scanner (3DHistech Ltd., Budapest, Hungary) and viewed on CaseViewer version 2.3.

### Mouse xenograft tumor model

All animal experimental procedures were performed in accordance with protocols approved by the Institutional Animal Care and Research Advisory Committee of Shandong Provincial Hospital Affiliated to Shandong University. Specific pathogen-free (SPF)-grade 5-week-old female severe combined immunodeficiency (SCID) beige mice (*n* = 6 per group) were housed in individually ventilated cages. The in vivo tumor growth study was performed as previously described [20]. A total of 1 × 10^7^ LY1 cells resuspended in 100 μl PBS mixed with 100 μl Matrigel (Corning, MA, USA) were subcutaneously injected into the flanks of mice. Tumor size was measured with a digital caliper. For in vivo therapeutic studies with AG1024, SCID beige mice were injected subcutaneously with 1 × 10^7^ LY1 cells (resuspended in 100 μl PBS mixed with 100 μl Matrigel) in the left inferior limb. One week later, the mice were blindly randomized and treated with daily intraperitoneal injections of AG1024 (30 μg/day), or vehicle control for 10 days (*n* = 6 per group). Tumor dimensions were measured every 2 days, and tumor volumes were calculated using the equation *V* = (*l* × *w*^2^) × 0.5, where *l* is the largest dimension and *w* is the perpendicular diameter.

### Statistical analysis

Data are represented as the mean ± standard deviation (SD) from at least three separate experiments. Differences between groups were analyzed by one-way analysis of variance (ANOVA) or *t* tests. Overall survival time was measured from the date of diagnosis to the date of death or last follow-up. Survival analyses were performed using the Kaplan-Meier method, and the log-rank test was used to identify significant differences. Univariate and multivariate analyses were performed using the Cox proportional-hazards regression model. All statistical analyses were performed with SPSS Statistics version 20.0 and GraphPad Prism version 6.0 statistical software. *P* < 0.05 was considered statistically significant.

## Results

### YAP expression is elevated in DLBCL and positively associated with disease progression

**Fig. 1:**
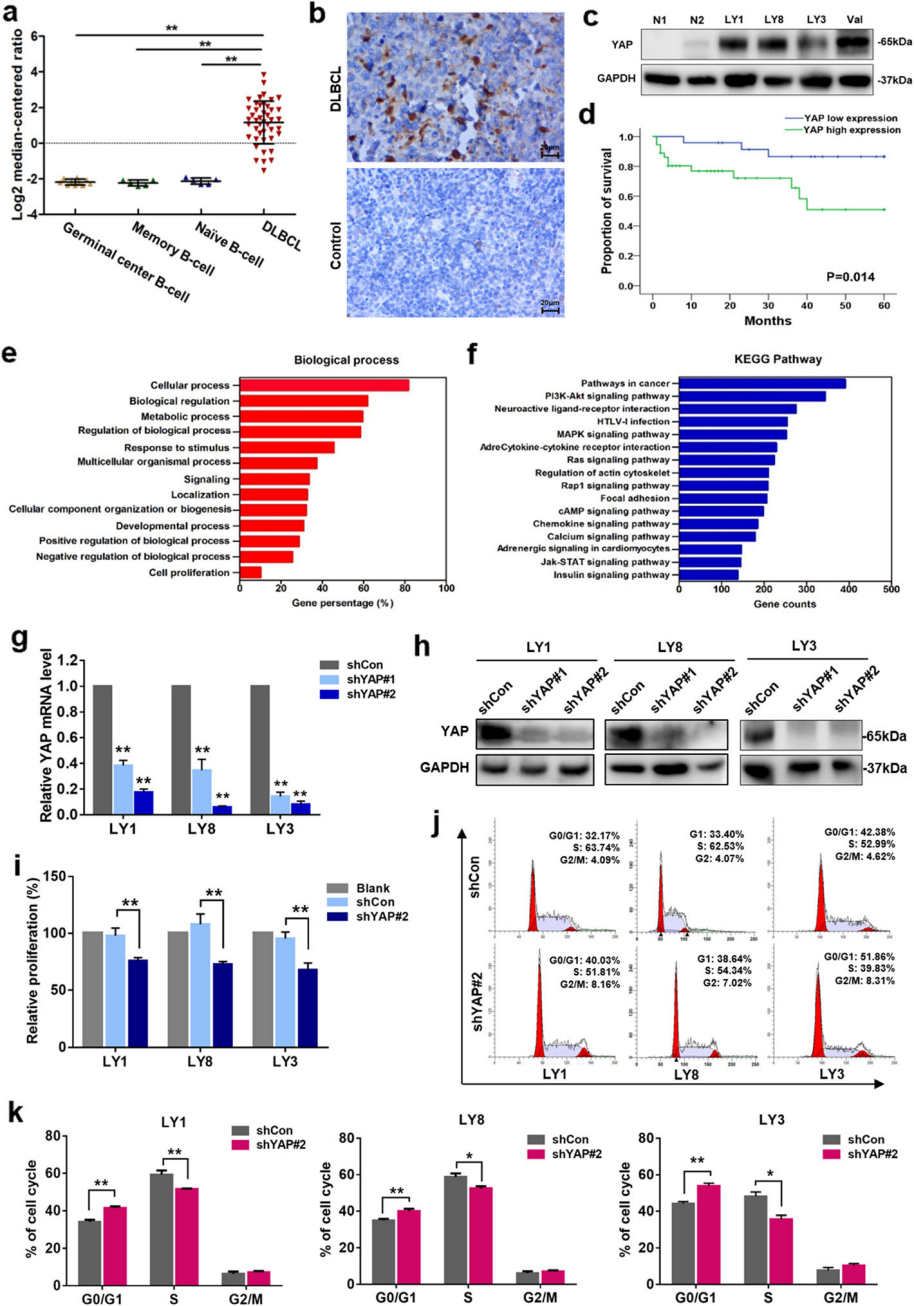
YAP is overexpressed in DLBCL and promotes cell proliferation. **a** The relative ratio of YAP mRNA in DLBCL tissue samples versus that in normal B cells in the Oncomine database. **p < 0.01. **b** Immunohistochemical staining for YAP in DLBCL primary samples and reactive lymphoid hyperplasia specimens. One representative stained sample is shown for each group. Bar = 20 μm. **c** Western blot analysis of YAP protein expression in DLBCL cell lines and normal B cells. **d** Analysis showing that DLBCL patients with high YAP expression presented significantly shorter survival times than those with low YAP expression. **e**, **f** GO and KEGG enrichment analysis of YAP expression in DLBCL microarray profiles. **g** Quantitative real-time PCR analysis of YAP mRNA expression in LY1, LY8, and LY3 cells after YAP knockdown compared to that in negative control cells. Data are presented as the mean ± SD from three independent experiments. **p < 0.01. **h** Expression of the YAP protein assessed by western blot analysis. **i** Relative proliferative levels of LY1, LY8, and LY3 cells transfected with shYAP or shCon detected by CCK-8 assay. Data are shown as the mean ± SD of at least three independent experiments. **p < 0.01. **j**, **k** Representative results for the cell cycle distributions of LY1, LY8, and LY3 cells with YAP knockdown. Data are shown as the mean ± SD. *p < 0.05, **p < 0.01

To elucidate the potential role of YAP in human cancers, we first examined the expression of YAP in data from the Oncomine database [24]. YAP expression levels were upregulated (tumor versus normal) in 6 out of 29 lymphoma datasets using the threshold of > 2-fold change and *p* value < 0.0001 (Figure S1). We next analyzed the microarray datasets [25] obtained from the Oncomine database to illuminate the YAP mRNA transcriptional alterations between normal B cells and DLBCL samples. As shown in Fig. 1a, the mRNA level of YAP was significantly elevated in the DLBCL tissue samples (*p* < 0.01). To assess the protein expression level of YAP in DLBCL patients, YAP expression was detected by IHC in a cohort of DLBCL primary samples (*n* = 60) diagnosed at Shandong Provincial Hospital Affiliated to Shandong University. Compared to reactive lymphoid hyperplasia, DLBCL patients showed significantly higher levels of YAP (Fig. 1b). High YAP expression (YAP^high^) was detected in 60% (36/60) of the DLBCL primary samples but only 23.3% (7/30) of the reactive lymphoid hyperplasia tissue samples (*p* = 0.001). Upregulation of YAP expression was validated in DLBCL cell lines. Consistently, the YAP expression level was significantly higher in human DLBCL cell lines than in normal B lymphocytes (Fig. 1c).

To address the clinical significance of YAP upregulation in DLBCL patients, the correlations between YAP expression and clinicopathological characteristics were analyzed. High levels of YAP expression were associated with B symptoms (*p* = 0.015), extranodal involvement (*p* = 0.023), and a high International Prognostic Index (IPI) score (*p* = 0.023) (Table 1), suggesting that upregulation of YAP expression was associated with DLBCL disease progression. Moreover, survival analysis of the enrolled patients revealed that higher expression of YAP was associated with a more aggressive disease process (*p* = 0.014) (Fig. 1d).

**Table 1:** Correlation between YAP protein expression and clinicopathologic parameters of the patients

Variables	No. of patients	YAP expression		***p*** value
		Positive	Negative	
**Age (years)**				
≤ 60	34	18 (52.9%)	16 (47.1%)	0.787
> 60	26	18 (56.2%)	14 (43.8%)	
**Gender**				
Male	30	19 (63.3%)	11 (36.7%)	0.598
Female	30	17 (56.7%)	13 (43.3%)	
**Ann Arbor stage**				
I or II	20	11 (55%)	9 (45%)	0.576
III or IV	40	25 (62.5%)	15 (37.5%)	
**B symptoms**				
Present	15	13 (86.7%)	2 (13.3%)	0.015*
Absent	45	23 (51.1%)	22 (48.9%)	
**Subtype**				
GCB	20	14 (70%)	6 (30%)	0.264
Non-GCB	40	22 (55%)	18 (45%)	
**Serum LDH**				
Normal	44	24 (54.5%)	20 (45.5%)	0.153
Elevated	16	12 (75%)	4 (25%)	
**Extranodal involvement**				
Absent	37	18 (48.6%)	19 (51.4%)	0.023*
Present	23	18 (78.3%)	5 (21.7%)	
**IPI score**				
0–2	37	18 (48.6%)	19 (51.4%)	0.023*
3–5	23	18 (78.3%)	5 (21.7%)	

*GCB* germinal center B cell-like, *LDH* lactate dehydrogenase, *IPI* International Prognostic Index

*p < 0.05

### Knockdown of YAP expression restrains cell growth and promotes cell cycle arrest

The above findings prompted us to further investigate the potential function of YAP in DLBCL. Functional enrichment analysis of YAP expression in DLBCL microarray profiles was performed. GO analysis indicated that YAP was closely related to cellular processes, biological regulation, and multicellular organismal processes (Fig. 1e). KEGG analysis revealed that YAP was enriched in pathways including cancer pathways and PI3K-Akt signaling (Fig. 1f). To validate the bioinformatics results, two lentivirus-mediated RNA interference vectors targeting YAP were used and exhibited remarkable silencing of YAP at the mRNA and protein levels in LY1, LY8, and LY3 cells (Fig. 1g, h), with shYAP#2 demonstrating higher efficacy. To examine the effect of YAP on the proliferation of DLBCL cells, a CCK-8 assay was performed. The results indicated that stable transfection of shYAP could significantly suppress the proliferation of DLBCL cells with high (LY1 and LY8) or low YAP expression (LY3) (*p* < 0.01) (Fig. 1i).

Flow cytometry was performed to evaluate the cell cycle distribution. The results demonstrated that knocking down of YAP expression induced a blockade in cell cycle progression at the G0/G1 phase (Fig. 1j, k). The apoptotic rates of DLBCL cells were also evaluated; however, no significant difference was detected after YAP knockdown. Therefore, silencing YAP restrained the growth of DLBCL cells predominantly through inhibition of cell proliferation and induction of cell cycle arrest.

### Targeted inhibition of YAP by VP exerts anti-tumor effects on DLBCL cells

**Fig. 2:**
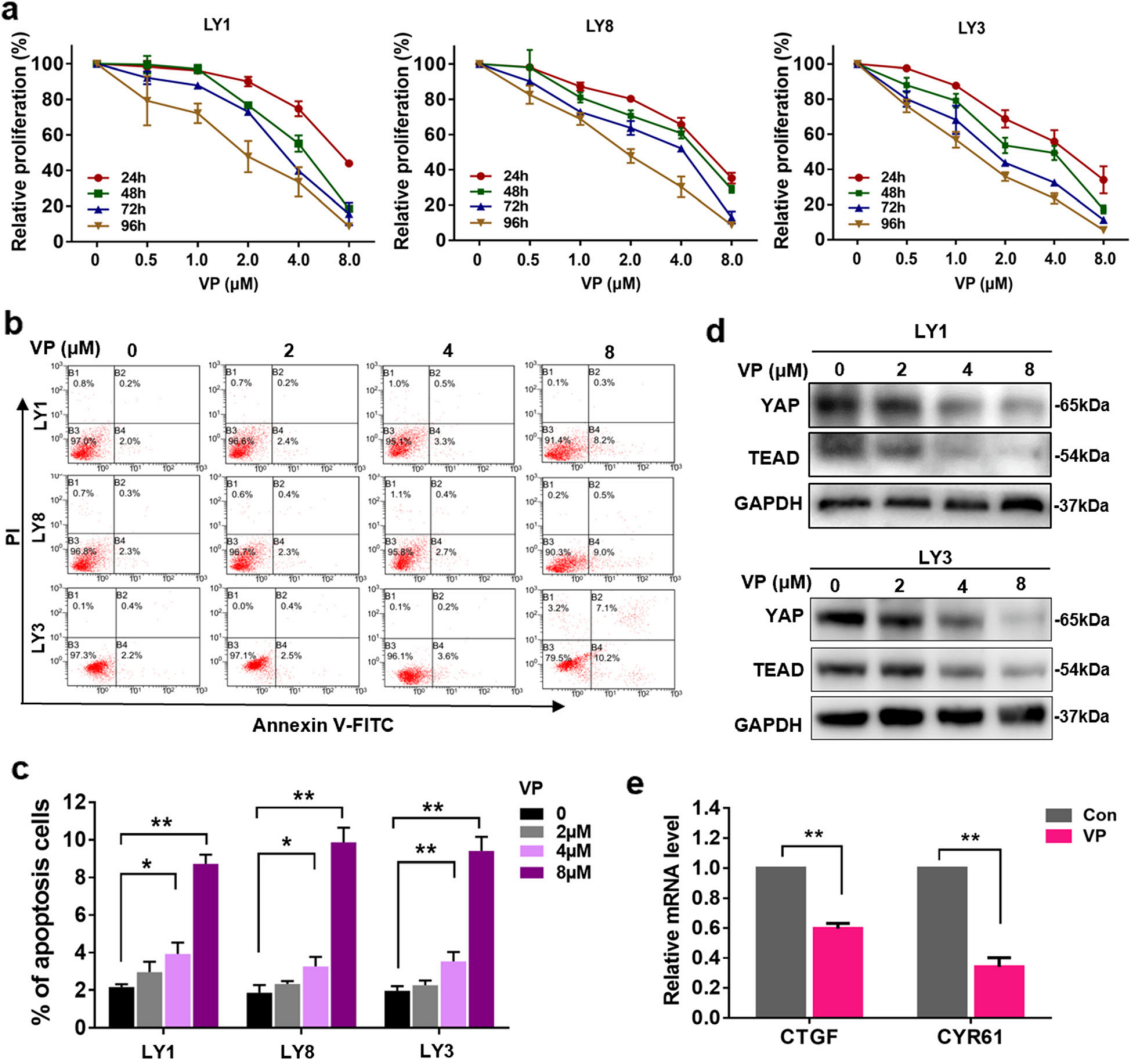
Targeted inhibition of YAP by VP exerts antitumor effects on DLBCL cells. **a** LY1, LY8, and LY3 cells were incubated with vehicle control or VP at various concentrations for 24, 48, 72, or 96 h. Relative cell proliferation was assessed with CCK-8. **b**, **c** Representative dot plots generated by flow cytometry analysis of LY1, LY8, and LY3 cells treated with VP for 24 h are shown. The results of the quantitative analysis are shown as the mean ± SD from at least three independent experiments. *p < 0.05, **p < 0.01. **d** The protein expression levels of YAP and TEAD were detected in LY1 and LY8 cells treated with VP at different concentrations. **e** The expression levels of YAP downstream targets were assessed by qRT-PCR. Data are presented as the mean ± SD from three independent experiments. **p < 0.01

To explore the function of YAP in DLBCL, we sought to examine the effect of YAP inhibition on DLBCL cells. VP, a Food and Drug Administration (FDA)-approved drug used as a photosensitizer for age-related macular degeneration [26], was shown to disturb the interaction between YAP and TEAD independent of light activation [27]. Accumulating evidence suggests that VP can inhibit YAP expression in several human malignancies [28, 29]. LY1, LY8, and LY3 cells were exposed to VP at the indicated concentrations for 24~96 h. We evaluated the effect of VP on cell viability in vitro and determined that the 50% inhibitory concentration (IC_50_) at 24 h for LY1 (7.172 ± 0.628), LY8 (5.687 ± 0.491), and LY3 (5.601 ± 1.027) cells aligned with the VP activity previously reported in other hematological malignancies [30]. Incubation with VP decreased the proliferation of DLBCL cells in a dose- and time-dependent manner (Fig. 2a). LY1 and LY3 cells treated with VP for 24 h were also subjected to apoptosis analysis by flow cytometry. As shown in Fig. 2b, c, compared to untreated cells, VP-treated DLBCL cells displayed robust apoptosis.

We next examined whether VP-induced apoptosis in DLBCL cells occurs through the regulation of YAP. As expected, decreased expression levels of YAP and TEAD were induced by VP in dose-dependent manner in LY1 and LY3 cells (Fig. 2d), indicating that the proapoptotic effect of VP on DLBCL cells occurs by abrogating the expression of YAP and TEAD. In addition, we found that treatment of VP (8 μM) strongly restrained the mRNA expression of YAP target genes CTGF and CYR61 (Fig. 2e). Taken together, these results demonstrate that VP restrains cell proliferation and induces cell apoptosis by inhibiting YAP expression.

### Deletion of YAP by CRISPR/Cas9 suppressed cell growth both in vitro and in vivo

**Fig. 3:**
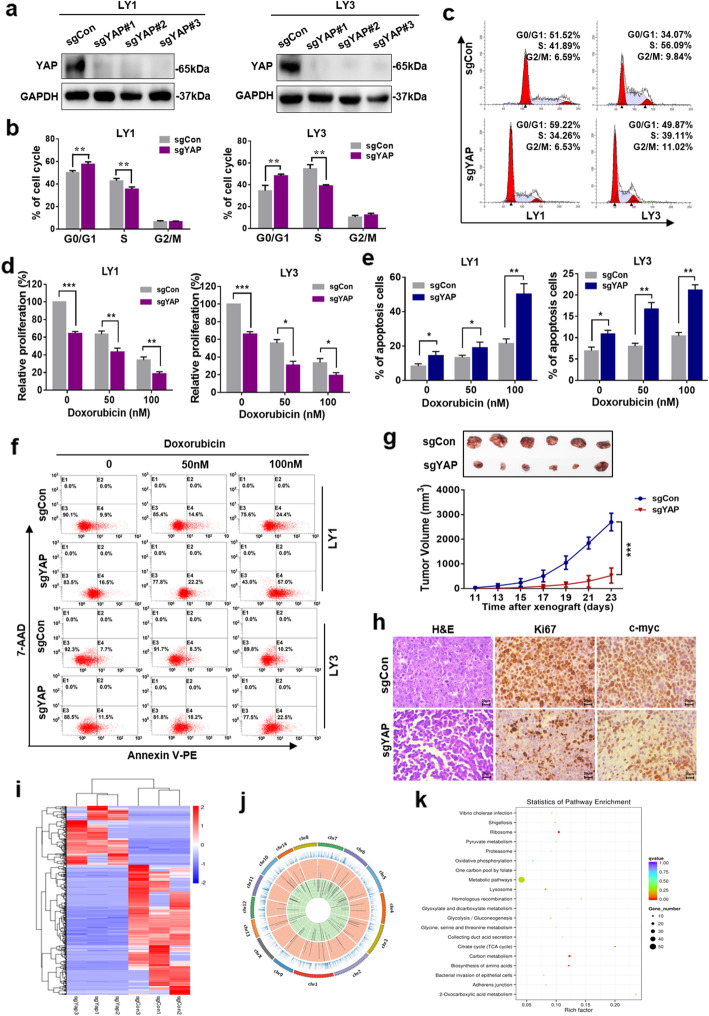
Deletion of YAP by CRISPR/Cas9 suppressed cell growth both in vitro and in vivo. **a** Western blot analysis confirmed CRISPR/Cas9-mediated YAP deletion in LY1 and LY3 cells. **b** Knocking out YAP induced cell cycle arrest in LY1 and LY3 cells, which were arrested in the G0/G1 phase. **p < 0.01. **c** Representative results for the cell cycle distribution analysis are shown. **d** LY1 and LY3 cells transfected with a control (sgCon) or sgYAP were treated with doxorubicin at the indicated concentrations for 24 h before being subjected to CCK-8 assay. *p < 0.05, **p < 0.01, ***p < 0.001. **e**, **f** Flow cytometry was used to analyze of cell apoptosis in LY1 and LY3 cells transfected with sgCon or sgYAP and treated with doxorubicin. *p < 0.05, **p < 0.01. **g** A xenograft DLBCL mouse model was established using LY1 cells with YAP knocked out. ***p < 0.001. **h** The expression levels of Ki67 and c-myc in xenograft tumors were determined. Bar = 20 μm. **i**, **j** The expression and location of differentially expressed (DE) mRNAs detected by RNA-seq in DLBCL cells with YAP knocked out were determined. **k** KEGG pathway analysis of DE molecules was performed

We further validated the involvement of YAP in DLBCL pathogenesis by deleting YAP with a CRISPR/Cas9 genome editing system in LY1 and LY3 cells. As indicated in Fig. 3a, three sgRNAs against YAP were designed. YAP deletion induced an increase in the proportion of DLBCL cells in the G0/G1 phase (*p* < 0.01) (Fig. 3b, c). Moreover, YAP^−/−^ cells showed a significant decrease in cell proliferation ability (Fig. 3d). We then tested the impact of YAP deletion on the response of DLBCL to a chemotherapeutic drug. Wild-type or YAP^−/−^ LY1 and LY3 cells were treated with doxorubicin, a chemotherapeutic reagent that is used to treat DLBCL. As shown in Fig. 3d–f, loss of YAP significantly enhanced the induction of growth inhibition and apoptosis by doxorubicin in these DLBCL cell lines.

A DLBCL xenograft mouse model was established with YAP^−/−^ LY1 cells to explore the effects of YAP inhibition in vivo. Compared to control tumors, tumors with YAP knocked out displayed a significantly reduced growth rate (Fig. 3g). Decreased expression levels of cell proliferation-related proteins, including Ki67 and c-myc, were also observed in tumors with YAP knocked out (Fig. 3h).

To further explore the mechanism underlying the transcriptional regulation of YAP in DLBCL, mRNA profiles were acquired by RNA-seq. A total of 528 mRNAs were shown to be significantly differentially expressed (DE) in YAP^−/−^ cells, with 364 downregulated and 164 upregulated. The most DE genes included RPL6 and RNF220 (Fig. 3i, j). Subsequent GO and KEGG pathway analyses revealed that the DE molecules were mainly related to cellular metabolic processes and carbon metabolism (Fig. 3k, Figure S2). We also detected changes in the expression of proteins involved in the proliferation-related mTOR and NF-κB pathways, such as LAMTOR1 and NFKBID (Figure S3), which was consistent with a previous study describing a multiprotein supercomplex involved in the control of oncogenic signaling in lymphoma [31].

### IGF-1R inhibitors exert cytotoxic effects on DLBCL cells

**Fig. 4:**
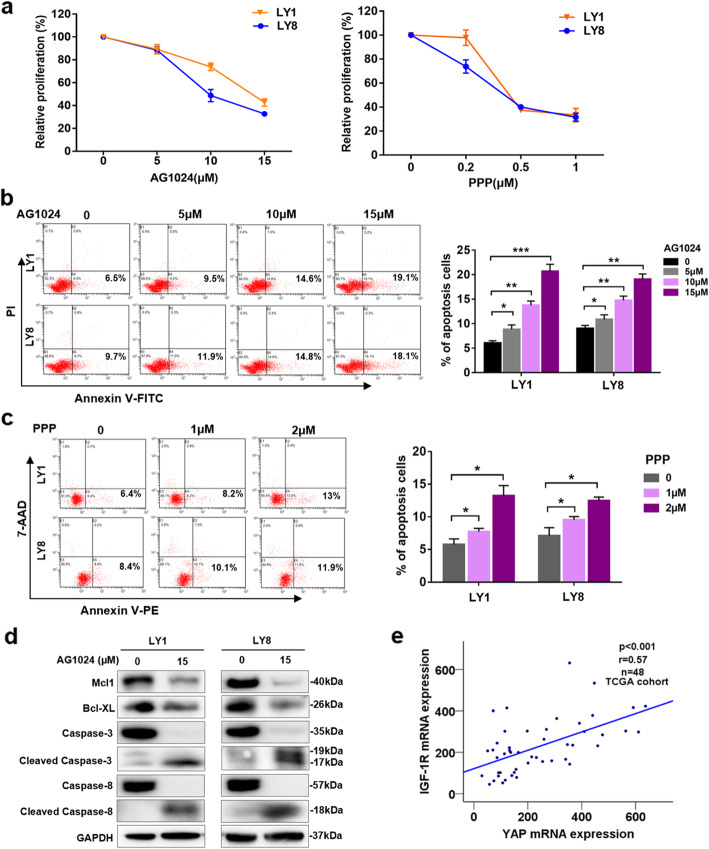
IGF-1R inhibitors exert cytotoxic effects on DLBCL cells. **a** LY1 and LY8 cells were treated with IGF-1R inhibitor AG1024 or PPP at the indicated concentrations for 24 h, and cell proliferation was determined by CCK-8 assay (mean ± SD, *n* = 6). **b**, **c** After treatment with an IGF-1R inhibitor (AG1024 or PPP) at the indicated concentrations for 24 h, cellular apoptosis was detected by flow cytometry. Representative results are shown. The results of the quantitative analysis are shown as the mean ± SD from at least three independent experiments. *p < 0.05, **p < 0.01, ***p < 0.001. **d**. Western blot analysis of DLBCL cell lines, treated with a single dose of 15 μM AG1024 for 24 h, was performed to determine the expression of the indicated proteins. **e** Scatter plots show the positive correlation between YAP and IGF1-R mRNA expression in the TCGA cohort

IGF-1R, a receptor tyrosine kinase (RTK), is implicated in the development and progression of malignant tumors [32]. Our previous study indicates that Klotho plays an antitumor role by regulating IGF-1R signaling [20]. Therefore, we hypothesized that targeting IGF-1R may have potential therapeutic value in DLBCL. DLBCL cell lines were treated with two structurally unrelated IGF-1R inhibitors, AG1024, a member of the tyrphostins molecule class that specifically inhibit the autophosphorylation of the tyrosin residue on IGF-1R [33], and PPP, a cyclolignan alkaloid that specifically inhibits the activity and downregulates the cellular expression of IGF1R without interfering with the activities of other growth factor receptors [34]. Either of these inhibitors could suppress the proliferation of DLBCL cells in a concentration-dependent manner (Fig. 4a). Flow cytometry was performed to determine the effects of IGF-1R inhibitors on the apoptosis of DLBCL cells. The apoptosis rates exhibited concentration-dependent increases following exposure to the IGF-1R inhibitors (Fig. 4b, c). Decreased expression levels of Mcl-1 and Bcl-XL, as well as cleavage of the apoptotic markers Caspase-3 and Caspase-8, were observed in LY1 and LY8 cells treated with AG1024 (Fig. 4d). These results show the antitumor effect of IGF-1R inhibitors on DLBCL.

To obtain insights into the function of IGF-1R in DLBCL, we stably transfected DLBCL cell lines with either IGF-1R-specific shRNAs (shIGF-1R) or an empty vector control (shCon). Transfection of shIGF-1R resulted in a significant reduction in cellular proliferation (*p* < 0.01) (Figure S4). Silencing IGF-1R significantly promoted cell apoptosis and downregulated Mcl-1 expression in DLBCL cells (Figure S4). In addition, the YAP mRNA level was positively correlated with IGF-1R in DLBCL samples deposited in The Cancer Genome Atlas (TCGA) database [35] (*r* = 0.57, *p* < 0.001) (Fig. 4e). Interestingly, the IGF-1R mRNA level was lower in DLBCL tissue samples than in normal tissue samples in the TCGA database (Figure S4). To clarify this, we examined the level of the active form p-IGF-1R in normal B cells and DLBCL cells. As shown in Figure S4, the p-IGF-1R level was upregulated in the DLBCL cells. This indicates that the active IGF-1R level in DLBCL cells is higher than that in normal B cells.

### Regulation of Hippo-YAP signaling by IGF-1R inhibitors

**Fig. 5:**
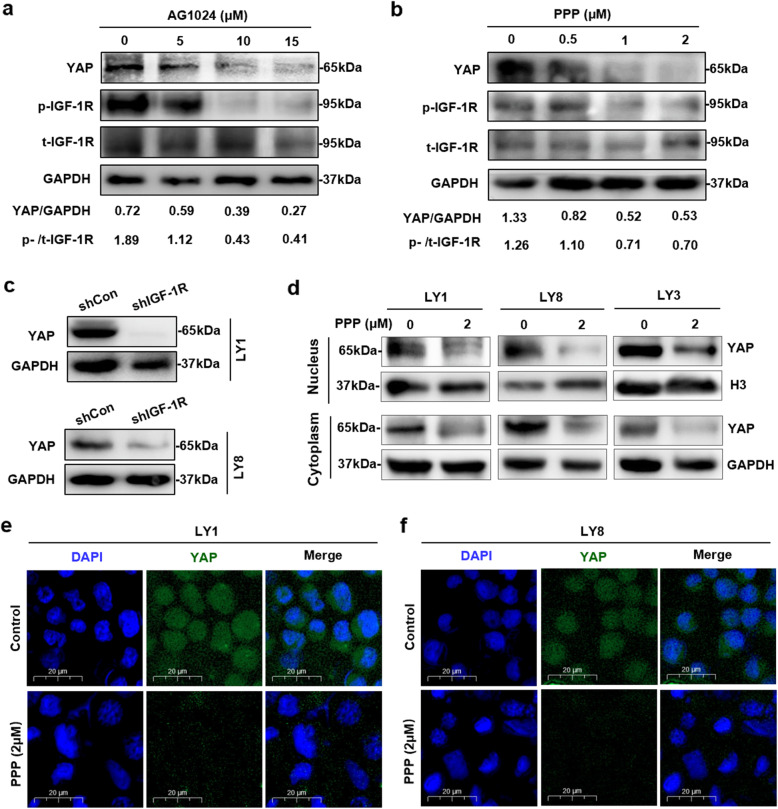
Regulation of Hippo-YAP signaling by IGF-1R inhibitors. **a**, **b** After treatment with the indicated concentrations of AG1024 or PPP for 24 h, LY1 cells were immunoblotted to determine the protein expression of YAP, p-IGF-1R, and t-IGF-1R. **c** Knockdown of endogenous IGF-1R reduced YAP protein expression in LY1 and LY8 cells. **d** LY1, LY8, and LY3 cells were treated with PPP (2 μM) for 24 h, and cytoplasmic and nuclear proteins were separated and extracted. YAP expression was detected by western blot analysis. **e**, **f** LY1 and LY8 cells were treated with PPP (2 μM) for 24 h, and immunofluorescence staining was performed to evaluate the endogenous expression and subcellular localization of YAP. Bar = 20 μm

We sought to explore the modulatory effect of IGF-1R on YAP expression in DLBCL. LY1 cells were incubated with various concentrations of IGF-1R inhibitors for 24 h. The results revealed that AG1024 and PPP significantly suppressed the phosphorylation of IGF-1R, which was accompanied by decreased YAP expression (Fig. 5a, b). To further validate the regulatory effect of IGF-1R on YAP expression, LY1 and LY8 cells were stably infected with either an shIGF-1R lentivirus or an shCon lentivirus. In accordance with the inhibitory effect of IGF-1R inhibitors, YAP expression was also significantly downregulated in LY1 and LY8 cells with IGF-1R knockdown (Fig. 5c).

The YAP level in the nucleus is modulated by the activity of Hippo signaling, and inactivated YAP is localized in the cytoplasm, where it is subsequently degraded. To further verify whether IGF-1R alters YAP activity by regulating the Hippo-YAP signaling pathway, we examined the effects of IGF-1R inhibitors on the subcellular expression of YAP in DLBCL cells by separating the nuclear and cytoplasmic proteins in DLBCL cells and detecting the subcellular expression of YAP protein. As shown in Fig. 5d, both the nuclear and cytoplasmic YAP levels were decreased, indicating increased inactivation and degradation of YAP following PPP treatment. This effect was confirmed by immunofluorescence assay (Fig. 5e, f). In addition, increased expression of MST1, a key protein in Hippo-YAP signaling, was observed in DLBCL cells treated with PPP or AG1024 (Figure S5). Enforced expression of MST1 was also detected in LY1 cells transfected with shIGF-1R (Figure S5). Collectively, these results indicate that IGF-1R depletion contributes to the modulation of Hippo-YAP signaling in DLBCL.

### Administration of IGF-1 induced YAP expression in DLBCL cells

**Fig. 6:**
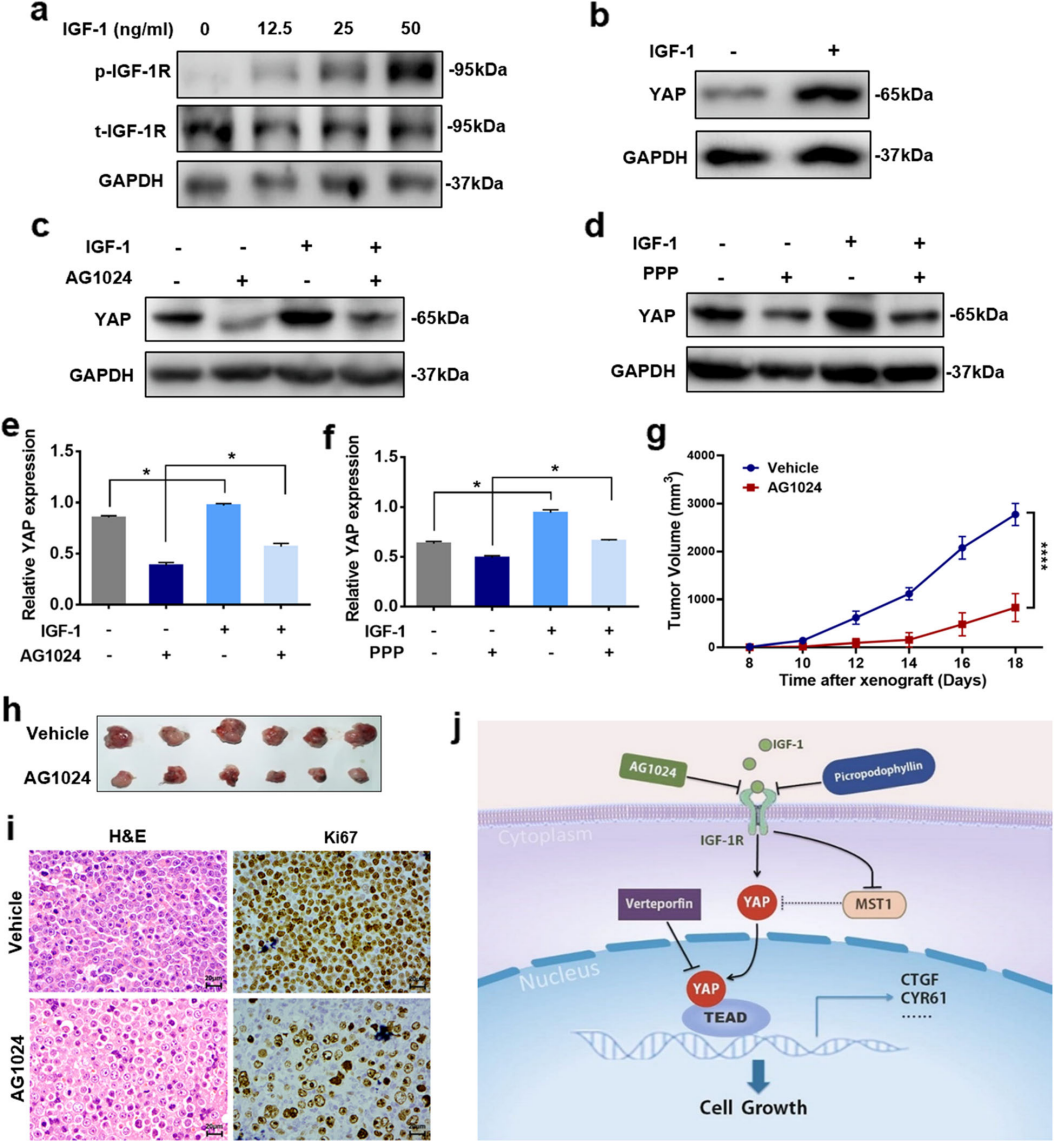
Administration of IGF-1 induced YAP expression in DLBCL cells. **a** LY1 cells were serum starved for 24 h and treated with IGF-1 (0 to 50 ng/ml) for 30 min. Western blotting was performed to determine the expression of p-IGF-1R and t-IGF-1R. **b** YAP expression was also assessed in LY1 cells treated with IGF-1(50 ng/ml). **c**–**f** IGF-1 rescued the expression levels of YAP which were decreased by the IGF-1R inhibitors AG1024 (15 μM) and PPP (0.5 μM), respectively. Quantitative data for the relative YAP protein levels are reported as the mean ± SD for triplicate experiments. *p < 0.05. **g**, **h** A mouse xenograft model was established with LY1 cells and treated with AG1024. Tumor volume was measured. ****p < 0.0001. **i** The Ki67 expression level was detected in the xenograft model. Bar = 20 μm. **j** The graphical representation summarizes the regulation of the Hippo-YAP pathway by IGF-1R

The above observations prompted us to confirm whether IGF-1 can rescue the decreased YAP expression caused by IGF-1R inhibition. Treatment with IGF-1 resulted in enhanced phosphorylation of IGF-1R in a dose-dependent manner (Fig. 6a). LY1 cells were serum starved for 24 h and treated with 50 ng/ml IGF-1 for 30 min. Western blot analysis was performed to evaluate the expression of YAP. As shown in Fig. 6b, IGF-1 induced the expression of YAP in LY1 cells. Additionally, IGF-1 rescued the decreased expression levels of YAP induced by AG1024 or PPP (Fig. 6c–f).

Based on these results, we sought to study the antitumor effect of AG1024 in DLBCL in vivo. A mouse DLBCL xenograft model was established and treated with AG1024. Compared to no treatment, AG1024 significantly inhibited tumor growth (Fig. 6g, h) and induced a decrease in Ki67 expression (Fig. 6i), indicating the great therapeutic potential of IGF-1R inhibitors in the treatment of DLBCL.

Overall, our results revealed the modulatory effect of IGF-1R on Hippo-YAP signaling in DLBCL (Fig. 6j).

## Discussion

Hippo-YAP signaling has been reported to be involved in several hematological malignancies, such as multiple myeloma, NK/T cell lymphoma, and leukemia [36–38]. In the current study, for the first time, we demonstrated the aberrant expression of YAP, a pivotal component of the Hippo-YAP signaling pathway, in DLBCL clinical specimens and cell lines. Elevated expression levels of YAP were correlated with aggressive disease and poor prognosis in DLBCL. VP diminished the proliferation of DLBCL cells by disturbing the expression of YAP and TEAD. Inhibition of IGF-1R resulted in dysregulated activation of Hippo-YAP signaling. Our findings demonstrate that IGF-1R may act as a critical upstream modulator of Hippo-YAP signaling.

The results of this study revealed that high expression of YAP was associated with a poor prognosis in patients with DLBCL. Further investigation of a larger sample will provide more comprehensive conclusions. Knocking down of YAP expression inhibited DLBCL cell growth mainly by inducing G0/G1 cell phase arrest in an expression-dependent manner. Muramatsu et al. [39] proposed that tumor cells overexpressing YAP exhibited a highly activated YAP-mediated pathway promoting proliferation. YAP may directly inhibit CDKN1A/p21 transcription to promote cell proliferation. Another possible explanation may be that activation of YAP protects cancer cells from DNA damage [40]. Our results provide evidence that the expression of YAP participates in the regulation of DLBCL initiation and progression.

Accumulating investigations have suggested that the Hippo-YAP signaling pathway and its effector, the YAP/TAZ-TEAD transcription complex, may provide potential targets for anticancer therapy [41]. Enhanced expression of YAP promotes proliferation and epithelial-mesenchymal transition in colorectal and prostate carcinomas [42, 43]. VP, a critical tool to illuminate the function of YAP in tumors, was shown to inhibit cancer cell growth in several types of human solid tumors [44–46]. Evidence has suggested that VP can inhibit cell proliferation and increase the efficacy of imatinib in chronic myeloid leukemia [30]. Our results demonstrated the potential therapeutic value of VP in DLBCL. Moreover, animal experiments will further confirm the biological effect and safety of VP as a DLBCL therapeutic strategy.

Notably, previous studies have indicated that oncogenic activation of RTKs contributes to the pathogenesis and progression of human malignancies. Constitutive activation of IGF-1R can induce ligand-independent neoplasm progression and contribute to the activation of identified oncogenes [47]. Strategies to block the IGF-1R pathway in solid malignancies, such as treatment with small molecular inhibitors or monoclonal antibodies, are being tested in clinical trials [48–50]. In this study, the significance of IGF-1R in DLBCL was verified through experiments with IGF-1R inhibitors performed in vitro. The proliferation of DLBCL cell lines was distinctly suppressed by IGF-1R inhibitors in a concentration-dependent manner. Moreover, IGF-1R inhibitors have been reported to modulate radiosensitivity and drug sensitivity in human solid tumors [51–53]. The combination of an IGF-1R inhibitor and chemotherapeutic drugs in relapsed/refractory solid tumors is currently being tested in several clinical trials [54].

Our results further illuminated that IGF-1R acted as an upstream negative regulator of Hippo-YAP signaling. Dysregulation of Hippo-YAP signaling in human malignancies mainly occurs via crosstalk with other signaling pathways involved in tumor formation. Recently, several transmembrane proteins, including epidermal growth factor receptor (EGFR) and G protein-coupled receptor (GPCR), have been reported to participate in the regulation of Hippo-YAP signaling; however, the detailed mechanisms remain to be clarified [55, 56]. It is extremely interesting to note that IGF-1R inhibition resulted in decreased activation of Hippo-YAP signaling in DLBCL. The interaction between IGF-1R and Hippo-YAP signaling has rarely been investigated. In the present study, we illustrated that the expression and nuclear accumulation of YAP in DLBCL could be significantly restrained by IGF-1R knockdown or IGF-1R inhibitor treatment. Moreover, in DLBCL cells, IGF-1 induced YAP expression and reversed the YAP downregulation induced by IGF-1R inhibitors. These results indicate that YAP may act as a downstream target of IGF-1R signaling in DLBCL, consistent with a previous study reporting the regulation of YAP by IGF-1R in liver cancer [57]. However, further investigations on the detailed molecular mechanisms involved in this process are still needed.

## Conclusions

In summary, our present data are the first to demonstrate the aberrant activation of Hippo-YAP signaling in DLBCL. YAP addiction may serve as a prognostic biomarker in DLBCL diagnosis. Loss of YAP function attenuates proliferation and induces cell cycle arrest in DLBCL cells. Given that inhibition of IGF-1R restrained YAP expression and showed anti-tumor effects on DLBCL cells, our findings indicate that targeting IGF-1R activity may produce therapeutic value in DLBCL by restricting YAP activity, which raises the possibility that molecular therapies targeting YAP will provide an attractive precise treatment strategy for DLBCL. Further investigation of the biological function of Hippo-YAP in DLBCL will highlight the crosstalk between these two pathways and outline a promising therapeutic option to utilize this newly identified oncogene in DLBCL therapy.

## Supplementary information

**Additional file 1: Table S1.** Sequences of primers used to amplify sgYAP cut sites.

**Additional file 2: Table S2.** Primer sequences for qRT-PCR.

**Additional file 3: Figure S1.** Analysis of the YAP expression profile in the Oncomine dataset. Summary of YAP mRNA expression in tissues samples from various human malignancies compared with corresponding normal tissue samples. Threshold: >2-fold change, *p*<0.0001. Red: upregulated in cancer; blue: downregulated in cancer.

**Additional file 4: Figure S2.** GO analysis of differentially expressed mRNAs detected by RNA-seq in DLBCL cells with YAP knocked out.

**Additional file 5: Figure S3.** mRNA expression levels of LAMTOR1 and NFKBID in YAP^-/-^ cells. Data were acquired from the RNA-seq analysis (***p<0.001, *p<0.05).

**Additional file 6: Figure S4.** Knockdown of IGF-1R expression inhibited the growth of DLBCL cells. **a.** LY1 and LY8 cells were treated with either sh-IGF-1R or shCon, and cell proliferation of cells was assessed by CCK-8 assay (**p<0.01). **b.** Flow cytometry assay indicated that treatment with shIGF-1R significantly induced cell apoptosis in DLBCL (**p<0.01). **c.** Decreased expression of Mcl-1 was detected in cells transfected with shIGF-1R. **d**. An analysis of data from TCGA database evaluated the mRNA levels of YAP and IGF-1R in DLBCL samples (*p<0.05). **e**. p-IGF-1R expression was increased in DLBCL cells compared to normal B cells. *The GAPDH blots are shared with **Figure****1****c**.

**Additional file 7: Figure S5.** Deficiency in IGF-1R leads to elevated MST1 expression in DLBCL cells. **a.** LY1 cells were treated with a single dose of 15 μM AG1024 for 24 h and immunoblotted for MST1. **b.** The expression of MST1 was assessed in LY1 cells treated with the indicated dose of PPP. **c.** Western blot analysis of MST1 in LY1 cells with IGF-1R knockdown was performed. **d.** The mRNA level of MST1 was determined in shCon and shIGF-1R cells from RNA-seq data (*p<0.05).

## Data Availability

The datasets used and/or analyzed during the current study are available from the corresponding author on reasonable request.

## Abbreviations

DLBCL: Diffuse large B-cell lymphoma
NHL: Non-Hodgkin lymphoma
YAP: Yes-associated protein
IGF-1R: Insulin-like growth factor-1 receptor
PBMCs: Peripheral blood mononuclear cells
IMDM: Iscove’s modified Dulbecco’s medium
VP: Verteporfin
PPP: Picropodophyllin
GFP: Green fluorescent protein
IHC: Immunohistochemistry
CCK-8: Cell Counting Kit-8
RNA-seq: RNA sequencing
GO: Gene ontology
KEGG: Kyoto Encyclopedia of Genes and Genomes
qRT-PCR: Quantitative real-time PCR
SPF: Specific pathogen-free
SCID: Severe combined immunodeficiency
SD: Standard deviation
OS: Overall survival
IPI: International Prognostic Index
FDA: Food and Drug Administration
DE: Differentially expressed
RTK: Receptor tyrosine kinase
shIGF-1R: IGF-1R-specific shRNAs
shCon: Empty vector control
EGFR: Epidermal growth factor receptor
GPCR: G protein-coupled receptor
