# Supplementary material for: Regulation of Hippo-YAP signaling by insulin-like growth factor-1 receptor in the tumorigenesis of diffuse large B-cell lymphoma
Source: J Hematol Oncol. 2020 Jun 16;13:77. doi: 10.1186/s13045-020-00906-1 (PMC7298789; doi:10.1186/s13045-020-00906-1)
Supplement: Supplementary file 1 — Additional file 1: Table S1. Sequences of primers used to amplify sgYAP cut sites. [file 13045_2020_906_MOESM1_ESM.docx]

**Table S1. Sequences of primers used to amplify sgYAP cut sites.**

| **Gene** | **Primer sequence** |
| --- | --- |
| **sgYAP#1** | F 5’- CACCGTGGGGGCTGTGACGTTCATC -3’ |
|  | R 5’- AAACGATGAACGTCACAGCCCCCA -3’ |
| **sgYAP#2** | F 5’- CACCGGAGCACTCTGACTGATTCTC -3’ |
|  | R 5’- AAACGAGAATCAGTCAGAGTGCTC -3’ |
| **sgYAP#3** | F 5’- CACCGACATCGATCAGACAACAACA -3’ |
|  | R 5’- AAACTGTTGTTGTCTGATCGATGT -3’ |

Abbreviations: F, forward primer; R, reverse primer
