# Supplementary material for: Regulation of Hippo-YAP signaling by insulin-like growth factor-1 receptor in the tumorigenesis of diffuse large B-cell lymphoma
Source: J Hematol Oncol. 2020 Jun 16;13:77. doi: 10.1186/s13045-020-00906-1 (PMC7298789; doi:10.1186/s13045-020-00906-1)
Supplement: Supplementary file 2 — Additional file 2: Table S2. Primer sequences for qRT-PCR. [file 13045_2020_906_MOESM2_ESM.docx]

**Table S2. Primer sequences for qRT-PCR.**

| **Gene** | **Primer sequence** |
| --- | --- |
| **YAP** | F 5’-TAGCCCTGCGTAGCCAGTTA-3’ |
|  | R 5’-TCATGCTTAGTCCACTGTCTGT-3’ |
| **CTGF** | F 5’-CTTGCGAAGCTGACCTGGAA-3’ |
|  | R 5’-AGCTCAAACTTGATAGGCTTGGAGA-3’ |
| **CYR61** | F 5’-CCAAGCAGCTCAACGAGGA-3’ |
|  | R 5’-TGATGTTTACAGTTGGGCTGGAA-3’ |
| **GAPDH** | F 5'-GGGAAACTGTGGCGTGAT-3' |
|  | R 5'-GAGTGGGTGTCGCTGTTGA-3' |

Abbreviations: F, forward primer; R, reverse primer
