# Supplementary material for: Regulation of Hippo-YAP signaling by insulin-like growth factor-1 receptor in the tumorigenesis of diffuse large B-cell lymphoma
Source: J Hematol Oncol. 2020 Jun 16;13:77. doi: 10.1186/s13045-020-00906-1 (PMC7298789; doi:10.1186/s13045-020-00906-1)
Supplement: Supplementary file 3 — Additional file 3: Figure S1. Analysis of the YAP expression profile in the Oncomine dataset. Summary of YAP mRNA expression in tissues samples from various human malignancies compared with corresponding normal tissue samples. Threshold: >2-fold change, p<0.0001. Red: upregulated in cancer; blue: downregulated in cancer. [file 13045_2020_906_MOESM3_ESM.docx]

**Figure S1**

**Disease Summary for YAP.**

**
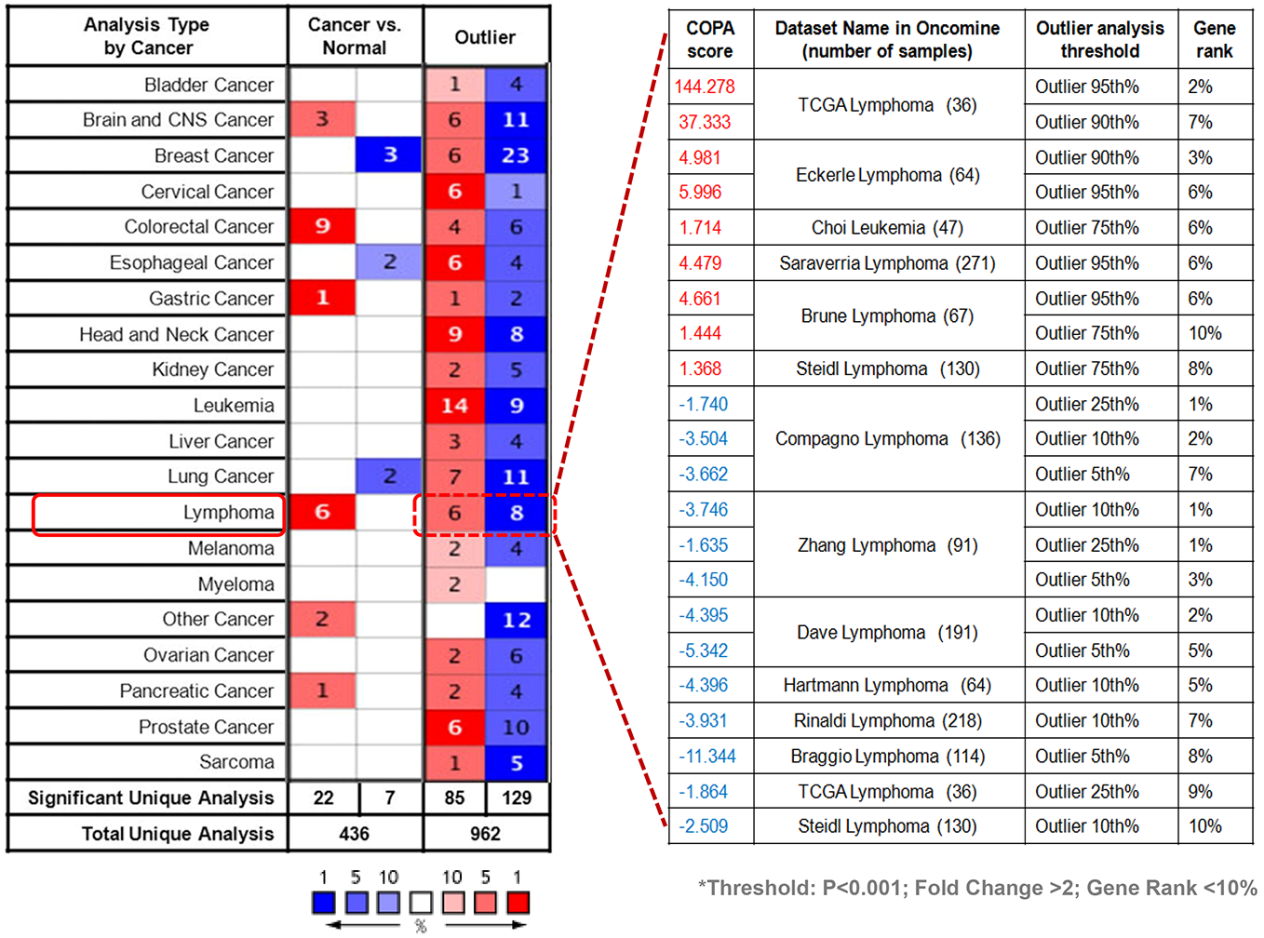
**

**Figure S1.** Analysis of the YAP expression profile in the Oncomine dataset. Summary of YAP mRNA expression in tissues samples from various human malignancies compared with corresponding normal tissue samples. Threshold: >2-fold change, *p*<0.0001. Red: upregulated in cancer; blue: downregulated in cancer.
