# Supplementary material for: Regulation of Hippo-YAP signaling by insulin-like growth factor-1 receptor in the tumorigenesis of diffuse large B-cell lymphoma
Source: J Hematol Oncol. 2020 Jun 16;13:77. doi: 10.1186/s13045-020-00906-1 (PMC7298789; doi:10.1186/s13045-020-00906-1)
Supplement: Supplementary file 4 — Additional file 4: Figure S2. GO analysis of differentially expressed mRNAs detected by RNA-seq in DLBCL cells with YAP knocked out. [file 13045_2020_906_MOESM4_ESM.docx]

**Figure S2**

**
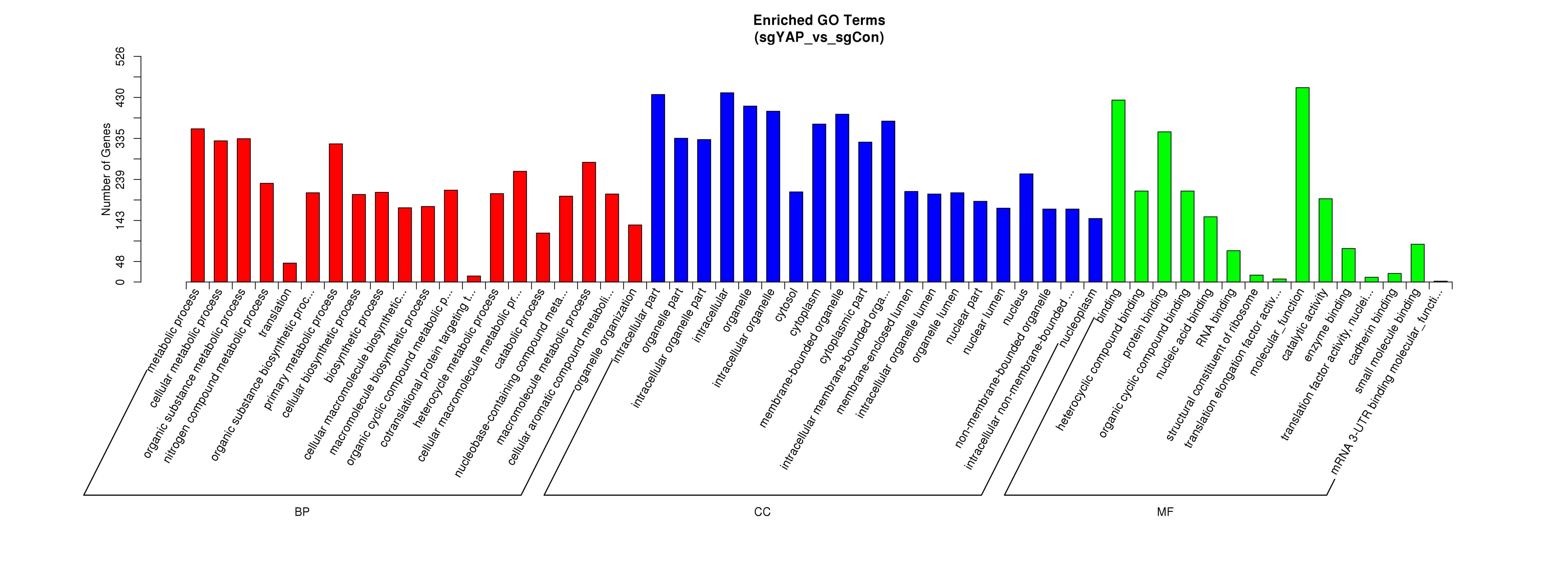
**

**Figure S2.** Gene ontology (GO) analysis of differentially expressed mRNAs detected by RNA-seq in DLBCL cells with YAP knocked out.
