# Supplementary material for: Regulation of Hippo-YAP signaling by insulin-like growth factor-1 receptor in the tumorigenesis of diffuse large B-cell lymphoma
Source: J Hematol Oncol. 2020 Jun 16;13:77. doi: 10.1186/s13045-020-00906-1 (PMC7298789; doi:10.1186/s13045-020-00906-1)
Supplement: Supplementary file 5 — Additional file 5: Figure S3. mRNA expression levels of LAMTOR1 and NFKBID in YAP-/- cells. Data were acquired from the RNA-seq analysis (***p<0.001, *p<0.05). [file 13045_2020_906_MOESM5_ESM.docx]

**Figure S3**

**
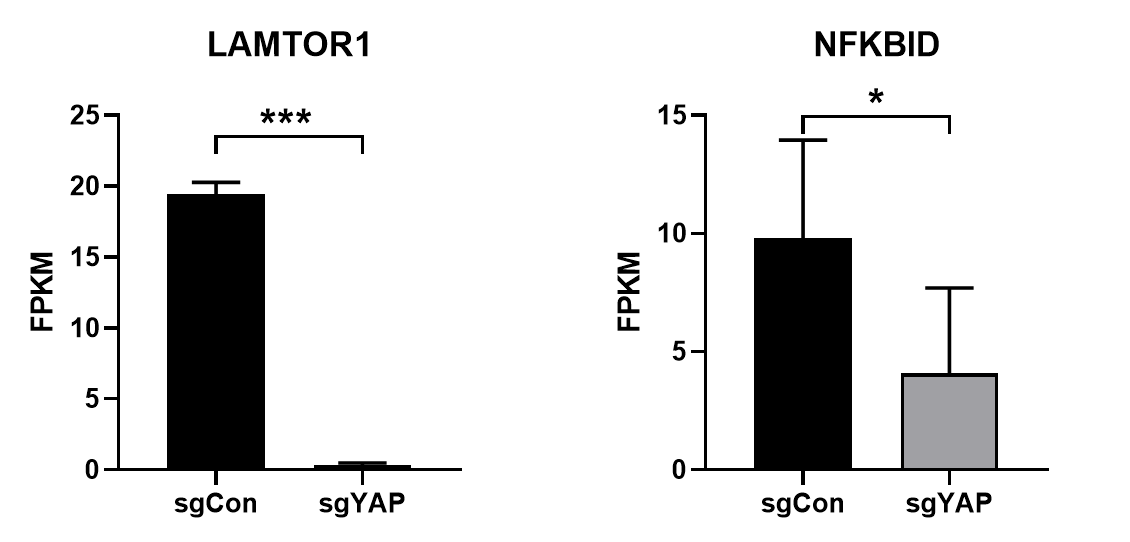
**

**Figure S3.** mRNA expression levels of LAMTOR1 and NFKBID in YAP^-/-^ cells. Data were acquired from the RNA-seq analysis (****p*<0.001, **p*<0.05).
