# Supplementary material for: Regulation of Hippo-YAP signaling by insulin-like growth factor-1 receptor in the tumorigenesis of diffuse large B-cell lymphoma
Source: J Hematol Oncol. 2020 Jun 16;13:77. doi: 10.1186/s13045-020-00906-1 (PMC7298789; doi:10.1186/s13045-020-00906-1)
Supplement: Supplementary file 6 — Additional file 6: Figure S4. Knockdown of IGF-1R expression inhibited the growth of DLBCL cells. a. LY1 and LY8 cells were treated with either sh-IGF-1R or shCon, and cell proliferation of cells was assessed by CCK-8 assay (**p<0.01). b. Flow cytometry assay indicated that treatment with shIGF-1R significantly induced cell apoptosis in DLBCL (**p<0.01). c. Decreased expression of Mcl-1 was detected in cells transfected with shIGF-1R. d. An analysis of data from TCGA database evaluated the mRNA levels of YAP and IGF-1R in DLBCL samples (*p<0.05). e. p-IGF-1R expression was increased in DLBCL cells compared to normal B cells. *The GAPDH blots are shared with Figure1c. [file 13045_2020_906_MOESM6_ESM.docx]

**Figure S4**


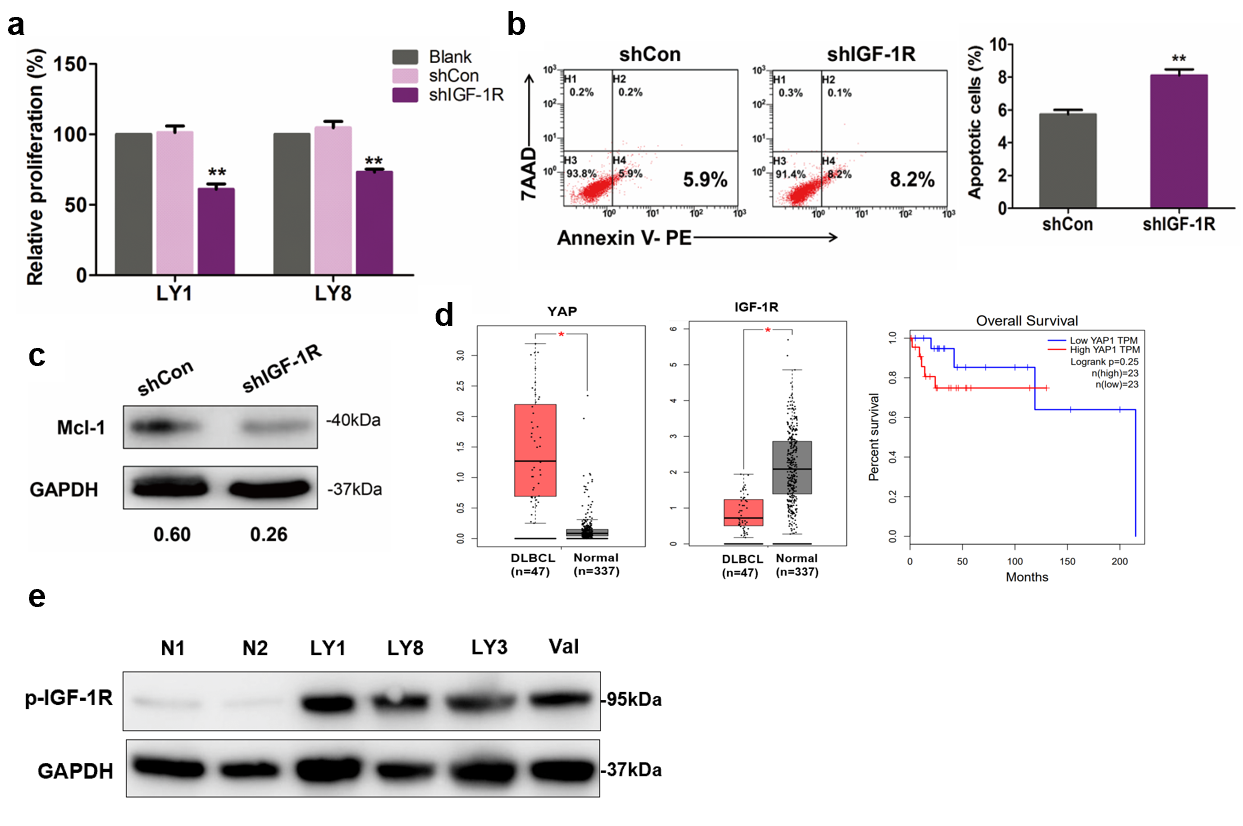


**Figure S4.** Knockdown of IGF-1R expression inhibited the growth of DLBCL cells. **a.** LY1 and LY8 cells were treated with either sh-IGF-1R or shCon, and cell proliferation of cells was assessed by CCK-8 assay (***p*<0.01). **b.** Flow cytometry assay indicated that treatment with shIGF-1R significantly induced cell apoptosis in DLBCL (***p*<0.01). **c.** Decreased expression of Mcl-1 was detected in cells transfected with shIGF-1R. **d**. An analysis of data from TCGA database evaluated the mRNA levels of YAP and IGF-1R in DLBCL samples (**p*<0.05). **e**. p-IGF-1R expression was increased in DLBCL cells compared to normal B cells. *The GAPDH blots are shared with **Figure 1c**.
