# Supplementary material for: Regulation of Hippo-YAP signaling by insulin-like growth factor-1 receptor in the tumorigenesis of diffuse large B-cell lymphoma
Source: J Hematol Oncol. 2020 Jun 16;13:77. doi: 10.1186/s13045-020-00906-1 (PMC7298789; doi:10.1186/s13045-020-00906-1)
Supplement: Supplementary file 7 — Additional file 7: Figure S5. Deficiency in IGF-1R leads to elevated MST1 expression in DLBCL cells. a. LY1 cells were treated with a single dose of 15 μM AG1024 for 24 h and immunoblotted for MST1. b. The expression of MST1 was assessed in LY1 cells treated with the indicated dose of PPP. c. Western blot analysis of MST1 in LY1 cells with IGF-1R knockdown was performed. d. The mRNA level of MST1 was determined in shCon and shIGF-1R cells from RNA-seq data (*p<0.05). [file 13045_2020_906_MOESM7_ESM.docx]

**Figure S5**

**
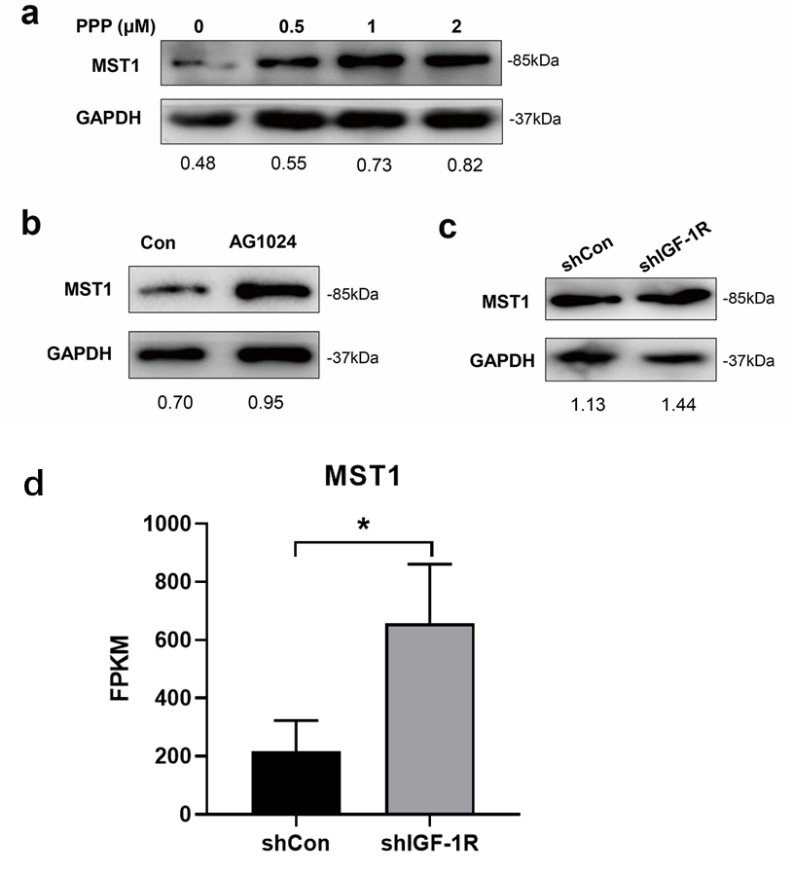
**

**Figure S5.** Deficiency in IGF-1R leads to elevated MST1 expression in DLBCL cells. **a.** LY1 cells were treated with a single dose of 15 μM AG1024 for 24 h and immunoblotted for MST1. **b.** The expression of MST1 was assessed in LY1 cells treated with the indicated dose of PPP. **c.** Western blot analysis of MST1 in LY1 cells with IGF-1R knockdown was performed. **d.** The mRNA level of MST1 was determined in shCon and shIGF-1R cells from RNA-seq data (*p<0.05).
